# A rare case of bilateral frontal lobe lesions due to thyroid storm

**DOI:** 10.20945/2359-4292-2023-0254

**Published:** 2024-04-22

**Authors:** Zhang Delong, Wang Fugui, Hu Xin, Lu Houqing

**Affiliations:** 1 Anhui Medical University Tongling Clinical College (Tongling People's Hospital) Intensive Care Unit Tongling China Intensive Care Unit, Tongling Clinical College (Tongling People's Hospital), Anhui Medical University, Tongling, 244000, China

## Abstract

Thyroid storm is a rare but well-known life-threatening complication that occurs due to acute exacerbation of thyrotoxicosis with the increased levels of circulating thyroid hormones. Reports of metabolic encephalopathy associated with thyroid storm are scarce. We describe the case of a 23-year-old male patient with no previous history of abnormal thyroid function who had consumed excessive amounts of alcohol before disease onset. The patient was found unconscious and febrile on a roadside by a passerby and was admitted to our hospital's emergency department. His primary clinical presentation included hyperthermia (40.8 °C), nodal tachycardia (180 beats/min), seizures, coma, and hypoglycemia (2.18 mmol/L). The hypoglycemia was quickly corrected after admission, but his level of consciousness showed no improvement. With aggressive screening, the patient was found to have severe thyroid dysfunction (T3 = 6.67 nmol/L, T4 = 252.00 nmol/L, free T3 = 29.20 pmol/L, free T4 = 65.30 pmol/L, and TSH = 0.001 μIU/mL). After medical treatment, plasmapheresis, hemofiltration, and hemoperfusion, the patient showed substantial improvement in thyroid hormone levels and stabilization of vital signs, but the impaired consciousness and seizures persisted. Multiple computed tomography scans revealed brain abnormalities. Magnetic resonance imaging performed after tracheal extubation revealed bilateral frontal lobe lesions. We reported a case of metabolic encephalopathy in a patient with life-threatening thyroid storm and bilateral frontal lobe lesions. Hypoglycemia may have been involved in the development of encephalopathy in our patient. Health care providers should consider thyroid storm in the differential diagnosis of hyperthermia, seizures, and coma. Early plasmapheresis, hemofiltration, and hemoperfusion can lower T4 levels and improve prognosis in patients with thyroid storm and encephalopathy.

## INTRODUCTION

Thyroid storm is a syndrome of acute exacerbation of thyrotoxicosis that occurs in association with increased levels of circulating thyroid hormones. It occurs mostly in patients with untreated or inadequately treated severe hyperthyroidism. Common triggers include infection, surgery, and mental stimulation. The clinical presentation of thyroid storm includes high fever, profuse sweating, tachycardia, irritability, anxiety, delirium, nausea, vomiting, and diarrhea; in severe cases, heart failure, shock, and coma may be present (1,2). The mortality rate associated with this complication is estimated to be between 8% and 25% despite modern advancements in treatment and supportive measures (3). To our knowledge, reports of metabolic encephalopathy associated with thyroid storm are rare.

Herein we present the case of a 23-year-old man without family history of thyroid disorders and no medical issues, who had ingested excessive amounts of alcohol before the onset of his condition. He was found unconscious and febrile on a roadside by a passerby and was admitted to our hospital's emergency department. Due to intense heat on the day of the patient's admission and his clinical presentation, we initially misdiagnosed him with heat apoplexy. However, the diagnosis of thyroid storm was established in a timely manner, and the patient was successfully treated.

After treatment, the patient presented substantial improvement in serum T4 levels and gradual stabilization of vital signs, but his level of consciousness and seizures persisted.

In this report, we discuss our experience treating this patient and describe the occurrence of metabolic encephalopathy found in association with his thyroid storm.

## CASE PRESENTATION

A 23-year-old man was brought to the emergency room of our hospital with hyperthermia, nodal tachycardia, seizures, and coma. He had a body temperature of 40.8 °C, heart rate of 180 bpm, blood pressure of 143/90 mmHg, and a respiratory rate of 35 bpm with shallow breathing. On detailed history taking, we were informed that the patient had been out drinking since 6 pm the day before the admission and had not returned home all night. He was found unconscious on a roadside by a passerby at 6 am on the day of the admission. Because it was summer and the heat was intense, the first responders established a diagnosis of pyrexia. On physical examination, the patient was comatose with a score of 6 in the Glasgow Coma Scale (Eye = 2, Verbal = NT, Motor = 3). Both his eyes had sluggish pupillary reaction, and the neurological findings were negative. The muscle tone in the extremities was increased, and spasticity was observed. On the 2nd day of admission, the patient's father provided a more complete medical history but reported no other remarkable detail. The patient had no family history of thyroid, heart, or neurological diseases.

An initial arterial blood gas analysis of the patient revealed a pH of 7.32, PaO_2_ of 85 mmHg, PaCO_2_ of 36.2 mmHg, and standard base excess of −6.8 mmol/L. The results of serum biochemistry were the following: uric acid, 989 μmol/L; creatine kinase, 1.052 U/L; creatine kinase MB isoenzyme, 480 U/L; urea nitrogen, 7.5 mmol/L; creatinine, 65.0 μmol/L; and glucose 2.18 mmol/L (indicating hypoglycemia).

We established an initial diagnosis of pyrexia with rhabdomyolysis. Immediately after admission, hypoglycemia was corrected, and physical cooling was applied. Hemofiltration and hemoperfusion were started using the multiFiltrate Ci-Ca module with Fresenius Av600s Filters (Fresenius Medical Care GmbH, Bad Homburg, Germany) and JaFron HA380 disposable hemoperfusion cartridges (Jafron Biomedical Co., Zhuhai, China). Intravenous esmolol hydrochloride (Qilu Pharmaceutical Co., Jinan, China; State Medical Permit No. H19991059) was used to decrease the patient's heart rate. His fever improved only slightly, and his decreased level of consciousness and seizures persisted.

Thyroid function tests obtained on the second day of admission showed substantially abnormal results (T3 = 6.67 nmol/L, T4 = 252.00 nmol/L, free T3 = 29.20 pmol/L, free T4 = 65.30 pmol/L, and TSH = 0.001 μIU/mL). Based on these results, the diagnosis was changed to thyroid storm. We immediately started therapeutic plasma exchange using Plasmaflux P2 filters (Fresenius Medical Care), which has been used in patients with thyroid storm for quick removal of circulating thyroid hormones and decrease of other harmful plasma constituents (4,5). Antithyroid therapy was started promptly and included propylthiouracil (600 mg/day in divided doses administered every 6 hours) and iodine. Intravenous esmolol was infused continuously to control ventricular rate.

**Figure 1:**
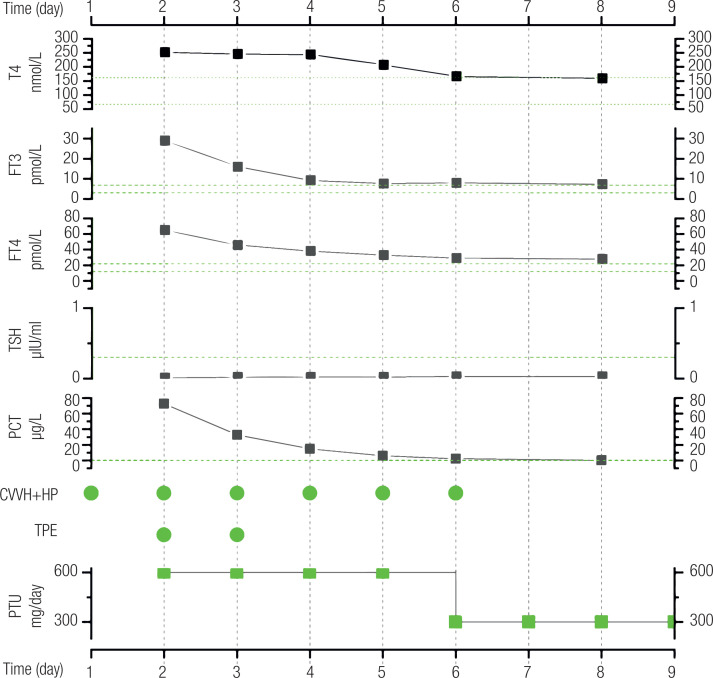
Primary management of the patient's thyroid storm and results of thyroid function tests and serum procalcitonin measurements. Abbreviations – CVVH+HP: continuous venous hemofiltration and hemoperfusion; FT3: free T3; FT4: free T4; PCT: procalcitonin; PTU: propylthiouracil; TPE: therapeutic plasma exchange. The green dots indicate the days when treatment was administered. The horizontal green dashed line indicates the reference range for the results.

The treatments started showing effect on the 3rd day, when the patient's tachycardia and body temperature improved substantially. Blood purification therapy, antithyroid drugs, hormonal therapy, and life support were maintained. These treatments helped the patient recover over the next few days. In total, two therapeutic plasma exchanges and six hemoperfusion cycles were performed. Gradually, the patient's heart rate and blood pressure returned to normal levels, and all thyroid function parameters showed improvement (Figure 1).

**Figure 2:**
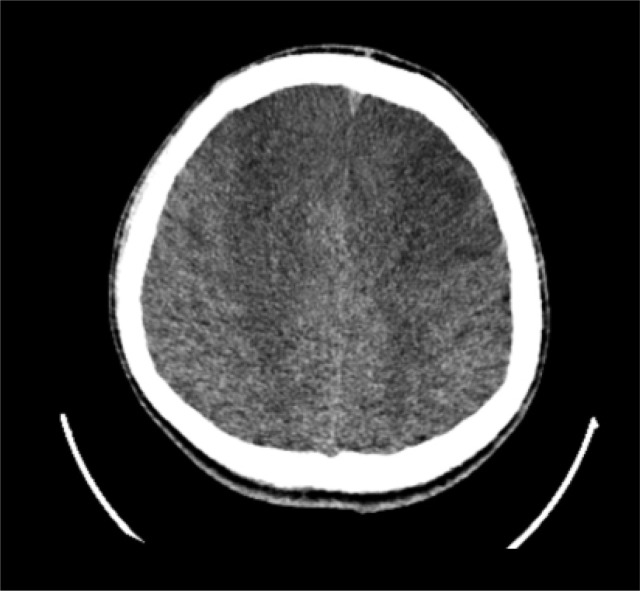
Computed tomography scan of the patient's brain obtained on the 8th day of admission.

**Figure 3:**
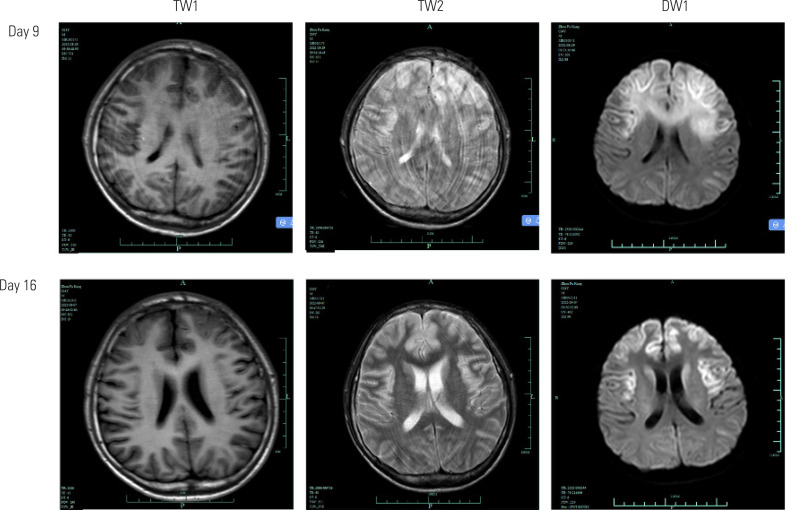
Results of magnetic resonance imaging (MRI) scans obtained on the 9th and 16th day of admission. Abbreviations – T1: T1-weighted sequences; T2: T2-weighted sequences; DWI: diffusion-weighted sequences.

On the 8th day of admission, all indicators of the primary disease were improving and the thyroid profile and other clinical parameters were as follows: T4 = 159 nmol/L, free T3 = 7.3 pmol/L, free T4 = 28 pmol/L, TSH = 0.03 μIU/mL, procalcitonin = 0.307 ug/L, temperature = 36.8 °C, heart rate = 87 bpm, blood pressure = 122/63 mmHg, and respiratory rate = 17 bpm. The patient was able to open his eyes spontaneously and follow sounds but was unable to move following verbal command. The seizures recurred on the 8th admission day. A computed tomography (CT) scan showed low-density bilateral frontal lobe lesions, but the nature of the lesions was unclear (Figure 2). On the 9th admission day, the patient's level of consciousness improved. He was weaned from the ventilator and underwent a magnetic resonance imaging (MRI) scan of the brain, which confirmed the presence of bilateral frontal lobe lesions (Figure 3). The lesions were not consistent with cerebral infarction; hence, we considered them to be caused by metabolic encephalopathy. We promptly arranged a hyperbaric oxygen chamber for rehabilitation. After that, the patient's level of consciousness improved further, and on the 16th day of admission we repeated the MRI scan, which showed substantial improvement of the bilateral frontal lobe lesions (Figure 3).

## DISCUSSION

Thyroid storm, also known as thyrotoxic crisis or thyroid crisis, is a life-threatening hypermetabolic thyrotoxicosis that presents as a multiorgan dysfunction with or without a known precipitating cause. It is a rare condition with an annual incidence of 0.57-0.76/100,000 persons in the general community and 4.8-5.6/100,000 persons hospitalized (6).

Among patients with untreated or poorly controlled hyperthyroidism, thyroid storm occurs in 1%-2% of the cases. It is commonly associated with Graves’ disease but may occur in association with toxic adenoma or toxic multinodular goiter (7).

The young patient in the present case had no previous thyroid disease. His initial diagnosis was obfuscated by the intense hot weather, the fact that he was an outdoor worker (intense physical exercise), and by his clinical presentation (coma, hyperthermia, increased heart rate, and absence of cerebral hemorrhage), leading to a misdiagnosis of heat apoplexy. The experience with the successful treatment administered to this patient indicates that health care providers must be vigilant with respect to intensive care medical treatment. Even the most typical clinical manifestations should not be ignored in the differential diagnosis. When a patient with hyperthermia and impaired consciousness is admitted to the emergency room, the following diseases (along with their associated factors and management) should be considered first:

- Heat apoplexy: high temperature and high humidity in the environment, high intensity exercise, shortness of breath, hyperthermia, and cramps.
- Hypertensive cerebral hemorrhage: cerebrovascular accidents, which can present as febrile episodes with altered level of consciousness and respiratory distress.
- Central nervous system infection: analysis of cerebrospinal fluid, in addition to physical examination, must be performed to identify meningeal irritation.
- Endocrine emergencies: diabetic ketoacidosis, thyroid storm, adrenal crisis.
- Malignant syndrome: use of antipsychotic drugs, mainly with a history of use of psychotropic drugs, muscle tone abnormalities, profuse sweating, elevated creatine kinase, and abnormal liver and kidney function.
- Severe dehydration: early diagnosis and early treatment are important factors to improve the prognosis of acute and critical illnesses.

The early misdiagnosis in the present case did not affect the symptomatic treatment, and intravenous esmolol hydrochloride, correction of hypoglycemia, physical cooling, and hemofiltration and hemoperfusion therapies were all necessary and effective. On the 2nd day of admission, we immediately initiated plasma exchange after establishing a definite diagnosis of thyroid storm, which quickly decreased the patient's T4 levels. Plasma exchange was indeed the main reason for the rapid control of his T4 levels. The patient had various life-threatening manifestations, coma, hyperthermia, tachycardia, and respiratory failure, indicating that we could not have waited for antithyroid therapy to take effect.

The persistent unconsciousness and seizures coupled with imaging findings in this patient led to the diagnosis of metabolic encephalopathy. This patient had two possible diagnoses to explain his metabolic encephalopathy. The first was hypoglycemic encephalopathy, as the patient had hypoglycemia (2.18 mmol/L) on admission with an unknown duration. The patient also had a history of excessive alcohol consumption, which could be related to the occurrence of hypoglycemia. The second was the diagnosis of hyperthyroid encephalopathy. Although only a few reports are available on encephalopathy caused by thyroid storm, no studies have been conducted to define the lesions.

Diffusion-weighted MRI of hypoglycemic encephalopathy shows symmetric hyperintense lesions in the cerebral cortex, hippocampus, and internal capsule (8). Since this patient had bilateral frontal lobe lesions, his presentation was not consistent with hypoglycemic encephalopathy. Additionally, clinical experience suggests that a blood glucose level of 2.13 mmol/L is not sufficient to cause severe hypoglycemic encephalopathy in a young patient. This patient developed metabolic encephalopathy after a thyroid storm, which we opted to define as a hyperthyroid encephalopathy caused by thyroid storm.

The patient was later diagnosed with Hashimoto's thyroiditis, which has been reported to cause Hashimoto's encephalopathy. The diagnosis of Hashimoto's encephalopathy is based on the following findings:

- Presence of neurological symptoms.
- Euthyroidism or mild hypothyroidism (9).
- Normal or non-specific MRI and cerebrospinal fluid findings (10).
- Elevated serum thyroid antibody (antithyroid peroxidase and/or antithyroglobulin) levels (11).

Subsequent next-generation sequencing and autoimmune antibody tests of the patient's cerebrospinal fluid also ruled out the diagnoses of intracranial infection and autoimmune encephalitis. However, the patient did not fit the profile of Hashimoto's encephalopathy, as he had an acute thyroid storm as the primary presentation along with specific bilateral frontal lobe lesions. The mechanism of thyroid storm encephalopathy is unknown and may be related to the autoimmune antibodies involved in the pathogenesis of Hashimoto's thyroiditis. Notably, several thyroid-related antigens are expressed in the brain, including thyroid peroxidase, thyroglobulin, thyrotropin receptor, myelin oligodendrocyte glycoprotein, tumor cell antigens, alpha-enolase, gangliosides, and other enzymes.

It is also possible that the patient's excessive alcohol consumption led to systemic vasodilatation. Since thyroid storm substantially increases the brain metabolism, the ensuing shock led to uneven cerebral perfusion, and the combined effects of hyperthyroidism and hypoglycemia resulted in hypoxic-ischemic changes in the bilateral frontal lobes. Metabolic encephalopathy after thyroid storm is rare, and we believe that the presence of hypoglycemia in this case may have been an important synergistic factor.

In conclusion, we reported a case of metabolic encephalopathy in a patient with life-threatening thyroid storm and bilateral frontal lobe encephalopathy with possible involvement of hypoglycemia. Health care providers must be attentive to the importance of including thyroid storm in the differential diagnosis of hyperthermia, seizures, and coma. Early plasmapheresis, hemofiltration, and hemoperfusion can lower T4 levels and improve prognosis in these patients.
